# Unique Function in Cancer Stemness and Prognostic Significance of *EMX2* in Esophageal Squamous Cell Carcinoma

**DOI:** 10.3390/biomedicines13061373

**Published:** 2025-06-04

**Authors:** Shoichi Fumoto, Keiji Tanimoto, Takuya Noguchi, Jun Hihara, Eiso Hiyama, Keiko Otani, Megu Ohtaki, Yutaka Shimada, Masahiko Nishiyama, Keiko Hiyama

**Affiliations:** 1 Department of Translational Cancer Research, Research Institute for Radiation Biology and Medicine, Hiroshima University, Hiroshima 734-8553, Japan; sfumoto@oita-u.ac.jp (S.F.); nogutaku@rg7.so-net.ne.jp (T.N.); m.nishiyama@gunma-u.ac.jp (M.N.); 2 Department of Gastroenterological Surgery, Oita Nakamura Hospital, Oita 870-0044, Japan; 3 Department of Gastroenterological Surgery, JA Oita Kouseiren Tsurumi Hospital, Oita 874-8585, Japan; 4 Department of Surgical Oncology, Research Institute for Radiation Biology and Medicine, Hiroshima University, Hiroshima 734-8553, Japan; j-hihara@asa-hosp.city.hiroshima.jp; 5 Hiroshima City North Medical Center Asa Citizens Hospital, Hiroshima 731-0293, Japan; 6 Natural Science Center for Basic Research and Development, Hiroshima University, Hiroshima 734-8553, Japan; eiso@hiroshima-u.ac.jp; 7 Department of Environmetrics and Biometrics, Research Institute for Radiation Biology and Medicine, Hiroshima University, Hiroshima 734-8553, Japan; ohtani@hiroshima-u.ac.jp (K.O.); ohtaki@hiroshima-u.ac.jp (M.O.); 8 Department of Nanobio Drug Discovery, Graduate School of Pharmaceutical Sciences, Kyoto University, Kyoto 606-8501, Japan; shimada.yutaka.3s@kyoto-u.ac.jp; 9 Division of Integrated Oncology Research, Gunma University Initiative for Advanced Research, Gunma University, Maebashi 371-8511, Japan

**Keywords:** ESCC, prognostic marker, cancer stemness

## Abstract

**Background/Objective:** The Empty Spiracles Homeobox 2 (EMX2) gene is a homeobox transcription factor that is critical for the development of the central nervous system and genitourinary system during embryogenesis. EMX2 has been shown to regulate cellular differentiation, migration, and proliferation through its involvement in transcriptional control. Dysregulation of EMX2 expression has been implicated in various pathological conditions, including cancer, but the precise molecular mechanisms underlying EMX2 functions in cancer remain incompletely understood. In this study, we focus on the expression profile and the prognostic significance of EMX2 in esophageal squamous cell carcinoma (ESCC). **Methods/Results:** The expression levels of *EMX2* in clinical ESCC samples varied and appeared to be lower than those in adjacent normal tissues. In addition, *EMX2* expression was detected in some of the 20 ESCC cell lines but not in others and was correlated with 5-FU sensitivity. *EMX2* expression in ESCC cell lines was strongly associated with colony formation capacity in soft agar, and *EMX2* knockdown decreased colony formation. Enforced expression of *EMX2* decreased the side population (SP) ratio in FACS analysis but increased colony formation in SP fractions. Although it is a preliminary experiment, xenograft in immunodeficient (NOD) *scid* mice suggested that the forced expression of EMX2 increased tumorigenic capacity in vivo. A Kaplan–Meyer analysis of patients from whom 20 ESCC cell lines or 18 ESCC tissue samples were obtained indicated that *EMX2* expression was a poor prognostic marker. **Conclusion:** EMX2 has a unique function in ESCC stemness and its expression is the stamped marker of poor prognosis in ESCC patients.

## 1. Introduction

Esophageal squamous cell carcinoma (ESCC) is a major subtype of esophageal cancer and is one of the leading causes of cancer-related mortality worldwide, particularly in East Africa, parts of South America, and East Asia, including Japan. Despite advances in surgical techniques, chemotherapy, and radiotherapy, the prognosis for ESCC patients remains poor, with a 5-year survival rate of less than 20% in advanced cases [1,2]. The aggressive nature of this disease is largely due to late diagnosis, high recurrence rates, and resistance to conventional therapies. Recent advances in endoscopic imaging techniques, particularly the integration of hyperspectral imaging (HSI) with computer-aided diagnostic systems, have significantly improved the detection and classification of esophageal neoplasms. For example, the SAVE system enhances the visualization of cancerous lesions by simulating narrow-band imaging (NBI) from conventional white-light images, thereby improving diagnostic precision without requiring specialized equipment [3]. Similarly, a CAD system that incorporates HSI and deep learning has achieved up to an 8% improvement in diagnostic accuracy compared to conventional RGB-based models [4]. These imaging innovations underscore the importance of identifying early tissue alterations and reinforce the need to explore molecular markers that may be involved in the early pathogenesis of esophageal cancer. Therefore, improving clinical outcomes requires understanding the molecular mechanisms underlying ESCC progression and identifying biomarkers for prognosis and therapeutic targets.

We have previously investigated several chemosensitivity marker genes using an original statistical analysis of oligonucleotide microarray expression data based on a two-dimensional mixed normal model [5] for ESCC [6], ovarian cancer [7], and various malignancies [8]. During these studies, we found an interesting gene, Empty Spiracles Homeobox 2 (*EMX2*), as a critical prognostic factor in patients with ESCC. EMX2, a homeobox transcription factor, plays a critical role during embryonic development, particularly in the formation of the central nervous system and genitourinary system [9,10]. Homeobox genes, including EMX2, regulate transcriptional programs that control cellular differentiation, proliferation, and migration [11]. While the developmental roles of EMX2 are well documented, its functions in pathological conditions, particularly cancer, are not fully understood. Emerging evidence suggests that EMX2 may play a context-dependent role in tumorigenesis, acting as a tumor suppressor, depending on the tissue type and cellular environment. In several cancers, dysregulation of EMX2 expression has been implicated in altering cellular plasticity and tumor progression. For example, reduced EMX2 expression has been associated with poor prognosis in glioblastoma [12], lung adenocarcinoma, [13], and endometrial cancer [14], though mutations have not contributed to the risk of endometriosis [15]. These findings highlight the potential role of EMX2 in cancer biology. However, its specific functions and regulatory mechanisms in ESCC remain poorly characterized.

In this study, we investigated the expression profile and functional significance of EMX2 in ESCC. Analysis of clinical samples revealed variable expression of EMX2, with levels generally lower levels in tumor tissues compared to adjacent normal tissues. In addition, we investigated the relationship between EMX2 expression and tumor stemness by assessing its effect on colony formation in soft agar and side population (SP) cells, which are enriched for CSCs. Furthermore, the prognostic relevance of EMX2 was investigated by Kaplan–Meier survival analysis of both ESCC patient samples and cell lines. Our findings suggest that EMX2 expression is a marker of poor prognosis in ESCC and plays a unique role in regulating tumor stemness and chemotherapeutic sensitivity. These results not only advance our understanding of the biological functions of EMX2 but also highlight its potential as a prognostic biomarker and therapeutic target in ESCC.

## 2. Materials and Methods

### 2.1. Cell Culture 

The 20 KYSE human esophageal squamous cell carcinoma (ESCC) cell lines (KYSE-30, -140, -150, -170, -180, -200, -220, -350, -410, -450, -510, -520, -590, -770, -850, -890, -1170, -1190, -1250, and -2270) and non-cancerous esophageal epithelial cell samples, HEEC-1 and HKE-1, were prepared as previously described [16,17]. Other cell lines, nine lung cell lines (a small cell carcinoma PC-6 and its SN-38- and CPT-11-resistant variants SN2-5 and DQ2-2, respectively; adenocarcinomas PC-9 and PC-14 and their CDDP-resistant variants PC-9/CDDP and PC-14/CDDP; adenocarcinoma A549, and squamous cell carcinoma LC-S), four colon (COLO201, COLO320DM, HCC-48, and HCC-50), two gastric (MKN45 and HSC-42), two leukemia (K562, K562/DOX), six head and neck (KB, HSC2, HSC3, HSC4, KOSC2, and Ca9-22), one hepatocellular (HepG2), one cervical (HeLa), eleven breast (BT-20, BT-474, MCF7, MDA-MB-231, MDA-MB-361, MDA-MB-435, MDA-MB-453, MDA-MB-468, SK-BR-3, T-47D, and ZR751), ten ovarian (KF, SHIN3, KK, OVISE, OVTOKO, RMG-1, TAYA, SKOV3, KF28, and KFr13TX) cancer cell lines, and two mammary epithelial cells (MCF-12A and 184B5) were prepared as previously described [7,8,17]. All cancer cell lines were cultured in RPMI 1640 medium (NACALAI TESQUE, Inc., Kyoto, Japan) or DMEM/F12^TM^ (NACALAI TESQUE, Inc.) containing 10% heat-inactivated fetal bovine serum (FBS; BioWhittaker, Verviers, Belgium) at 37 °C in a humidified atmosphere of 5% CO_2_ and maintained in continuous exponential growth by passage every 3 days. Non-cancerous HEEC-1 and HKE-1 cells were cultured in Keratinocyte SFM^TM^ medium with growth supplement containing 2.5 mg EGF and 25 mg bovine pituitary extract in 500 mL liquid basal medium (Thermo Fisher Scientific K.K., Tokyo, Japan), 184B5 in MEGM^TM^ (CC-3150, Sanko Junyaku, Tokyo, Japan), and MCF-12A in DMEM/F12^TM^ (Invitrogen, Carlsbad, CA, USA). For gene expression analysis, exponentially growing cultured cells were harvested and stored at −80 °C until use.

### 2.2. Soft Agar Colony Formation Assay

Anchorage dependency of ESCC cell lines was evaluated by conventional colony formation assay with soft agar in triplicate, as previously reported [18]. Briefly, 5 × 10^3^ cells were cultured in 0.4% SeaPlaque GTG agarose (Lonza K.K., Tokyo, Japan), and after 14 days of culture at 37 °C with 5% CO_2_, colony number was counted microscopically (colonies containing more than 5 cells were counted) and confirmed macroscopically by crystal violet staining.

### 2.3. Patients and Surgically Resected Tissue Samples

A total of 42 ESCC tissue samples were obtained from chemo-naïve patients at the time of surgery. Of these, DNA and RNA from both tumor and adjacent non-cancerous tissues were available for this study in 17 cases and were used to compare *EMX2* expression levels between them. A total of 2 out of 17 cases showed poor-quality RNA in normal tissue, resulting in a comparison of 15 paired samples. Meanwhile, 18 patients were less than 80 years old (median: 61, range: 49–78) with World Health Organization (WHO) performance status 0 to 2 without significant baseline laboratory abnormalities, and life expectancy was estimated to be more than three months. The 5-FU was administrated by continuous intravenous infusion at a dose of 250 mg/m^2^/day for 28 days or as a 5-day continuous infusion of 500 mg/body/day per week for 28 days, in combination with cisplatin at a low dose of 3 mg/m^2^ or 10 mg/body/day. The total doses of 5-FU and CDDP administered ranged from 2625 to 10,500 mg (median 10,000 mg, mean 8912 mg) and from 26 to 200 mg (median 200 mg, mean 143 mg), respectively. Computed tomography (CT) scans were performed every one to two months to assess disease-free survival (DFS). Written informed consent was obtained from all patients, and the protocol was approved by our institutional ethics committees. The collected tumor samples were stored at −80 °C until use.

### 2.4. Microarray Analysis and Extraction of Candidate Genes Associated with Sensitivity to 5-FU

Microarray analyses were performed and described previously [6,16,17] using the CodeLink™ Expression Bioarray System (GE Healthcare, Tokyo, Japan) according to the manufacturer’s protocol. The microarray data were deposited in the Gene Expression Omnibus under GE accession no. GSE9982 (http://www.ncbi.nlm.nih.gov.geo/ access date: 1 December 2008). The candidate genes associated with sensitivity to 5-FU were extracted by the original statistical method, as previously reported [5]. Briefly, to filter out genes not associated with sensitivity to 5-FU, gene expressions in 20 ESCC cell lines analyzed by microarray were transformed by the functional states of a gene as “expressed/unexpressed”: if a gene is estimated to be unexpressed, its measurement is replaced by zero; otherwise the background corrected measurement is used. Pearson’s correlation coefficients and rank correlation coefficients were calculated between the IC_50_ values and the transformed gene expression levels. Their *p*-values were used to select the most predictive marker genes from the large number of candidates.

### 2.5. Quantitative RT-PCR

Total RNA (2 μg) extracted from each cell or tissue sample was reverse transcribed using the High-Capacity cDNA Archive^TM^ Kit (Applied Biosystems, Foster City, CA, USA). Two hundredth aliquots of the cDNA were subjected to quantitative RT-PCR (RT-qPCR) using the Universal Probe Library (UPL, Roche Diagnostics, Tokyo, Japan) for *EMX2sp1* and *EMX2sp2* (ENSG00000170370) (Supplementary Table S1), TaqMan^TM^ Gene Expression Assays (Applied Biosystems) for *PROM1* and *PCNA*, designed TaqManTM MGB probe designed for *TERT*, or an internal control *GAPDH* TaqMan^TM^ probe (Applied Biosystems). The reliability of *GAPDH* as an internal control was confirmed by comparing its expression level with that of *ACTB*, which was used as a second internal control, in all samples. The mRNA expression level of the target gene was calculated by the ratio to *GAPDH*, and the relative amount was standardized using a pooled cDNA derived from 17 different cancer cell lines, as previously described [17]. Each reaction was performed in triplicate using an ABI PRISMTM 7900 Sequence Detection System (Applied Biosystems).

### 2.6. Knockdown Analysis of EMX2 by siRNA

All siRNAs (Supplementary Table S2) were purchased from Thermo Fisher Scientific K.K. and transfected into an ESCC cell line with high *EMX2* expression (KYSE-350) using the siPORT^TM^ *NeoFX*^TM^ (Thermo Fisher Scientific K.K.) according to the manufacturer’s protocol.

### 2.7. Construction of the EMX2 Expression Plasmid Vector

Although *EMX2* has two isoforms, *sp1* and *sp2*, their expression levels were highly correlated, with the former always several times higher than the latter. We then constructed an *EMX2sp1* expression vector using cDNA derived from a normal fibroblast cell line MJ90 [19] as follows. The forward (F) and the reverse (R) primers contained *Eco*RI and *Bgl*II restriction sites, respectively (Supplementary Table S3). The PCR reaction mixture (20 μL) containing 0.2 μg of cDNA, 2 μL each of 2 mM dNTP, 0.3 μL each of 10 μM forward and reverse primers, and 0.4 U KOD-Plus^TM^ DNA polymerase (TOYOBO, Osaka, Japan) was subjected to an initial incubation at 94 °C for 2 min, followed by 38 cycles of denaturation at 94 °C for 40 s and annealing and extension at 68 °C for 2 min. The PCR product was digested with *Eco*RI and *Bgl*II restriction enzymes and ligated into the p3xFLAG CMV10 vector (Invitrogen). The sequence of the insert was confirmed by DNA sequencing (Applied Biosystems).

### 2.8. Transfection of EMX2 into ESCC Cell Lines

The *EMX2sp1*-expressing plasmid pEMX2-3xFLAG and control vector p3xFLAG-CMV10 were linearized by *Sca*I digestion and then transfected into two ESCC cell lines, KYSE-170 and -510, using *Trans*IT^®^-LT1 reagent (Mirus Bio Corporation, Madison, MI, USA) according to the manufacturer’s protocol followed by selection with G418.

### 2.9. Cell Sorting for Side Population (SP) and Main Population (MP)

Single-cell suspensions (10^6^ cells for each sample) incubated with 8 μM Hoechst 33342 (Invitrogen) at 37 °C for 90 minutes with or without 30 μg/ml of verapamil (Sigma-Aldrich, Inc., St. Louis, MO, USA) were rinsed with PBS containing 2% FBS, filtered with the Cell Strainer (Becton, Dickinson and Company, Flanklin Lakes, NJ, USA), mixed with 500 ng of propidium iodide (Becton, Dickinson and Company, Flanklin Lakes, NJ, USA), and subjected to FACSAria (Becton, Dickinson and Company, Flanklin Lakes, NJ, USA). The SP and MP fractions sorted through Hoechst red and blue filters were then subjected to soft agar colony formation assay.

### 2.10. Western Blotting

Whole-cell extracts were prepared from *EMX2*-expressing or control vector-transfected cancer cell lines using 100 μL/sample RIPA lysis buffer (Santa Cruz Biotechnology, Santa Cruz, CA, USA) and subjected to PAGE and blotting onto nitrocellulose filters as previously described [17]. A 1:2000 diluted anti-FLAG M2 (Sigma-Aldrich, Inc., St. Louis, MO, USA) and a 1:5000 diluted anti-mouse IgG-horseradish peroxidase conjugate (GE Healthcare, Tokyo, Japan) were used as primary and secondary antibodies, respectively, and visualized using the ECL-Plus^TM^ Western blotting system (GE Healthcare, Tokyo, Japan).

### 2.11. Transplantation into Non-Obese Diabetic (NOD) Scid Mice

Immunodeficient NOD *scid* mice were obtained from CLEA Japan (Tokyo, Japan). A total of 1 million and 10^3^ cells of *EMX2*-transfected (*n* = 4) and control vector-transfected (*n* = 2) ESCC cell clones, respectively, derived from two originally *EMX2*-absent ESCC cell lines, KYSE170 and KYSE510, were mixed with 100 µL of the Matrigel Matrix (Becton, Dickinson and Company, Flanklin Lakes, NJ, USA) and injected s.c. at a total of 18 sites (maximum of four loci per mouse) in the shoulder or flank of five mice. Tumor development was monitored until day 32. All work was performed under institutional animal care and use committee approval.

### 2.12. Statistical Analysis

The statistical analyses were performed in Figures 2, 3, 4, 6, and Figure S1 using StatView version 5.0 (SAS Institute Inc., Cary, NC, USA). Pearson or Spearman correlation tests were performed to confirm the correlation between genes, as appropriate. Survival analyses were performed using the Kaplan–Meier method with log-rank tests. *p*-values less than 0.05 were considered statistically significant.

## 3. Results

### 3.1. Expression Levels of EMX2 Evaluated by RT-qPCR in ESCC Tissue Samples and Various Cancer Cell Lines

**Figure 1:**
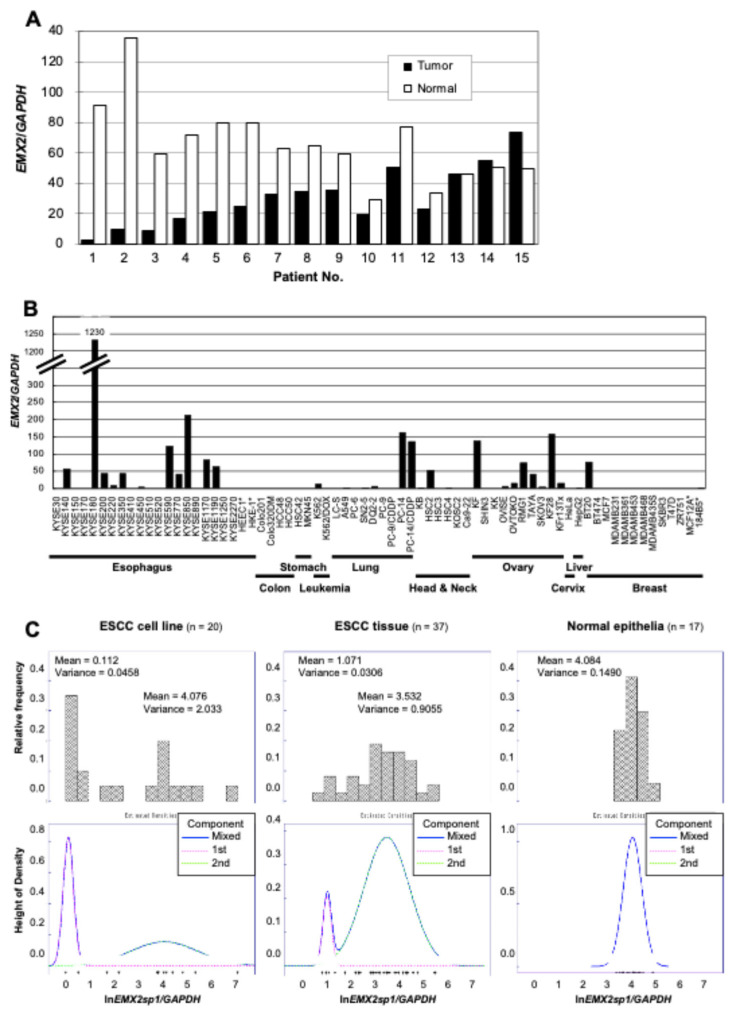
*EMX2* expression levels and their distribution in different cells and tissues evaluated by RT-qPCR. (**A**) *EMX2* expression in ESCC tissues (closed bar) and in the corresponding normal esophageal epithelium (open bar) was obtained from 15 patients who exhibited high-grade RNA in both normal and tumor tissues. Relative gene expression levels were calculated using *GAPDH* expression as the denominator for each sample (*n* = 3). (**B**) *EMX2* expression in different cancer cell lines and non-cancerous cells (*). Relative gene expression levels were calculated using *GAPDH* expression as the denominator for each sample (*n* = 3). (**C**) Distribution of *EMX2* expression in 20 ESCC cell lines (left), 37 ESCC tissues (middle), and 17 non-cancerous esophageal epithelia (right). Top panels show histograms of log-transformed *EMX2* expression levels. A log-likelihood ratio test was performed for each distribution to decide which model was more appropriate between a two-component mixed normal distribution and a one-component normal distribution. Bottom panels show estimated mixed normal density distributions of log-transformed *EMX2* expression levels. The pink and lime dotted lines show the first and second components, respectively, and the blue solid line shows the mixed distribution.

*EMX2* expression was detected in all tissue samples, and expression levels in 2/3 of ESCC tissues were lower than that in corresponding non-cancerous esophageal epithelia (*p* = 0.0028) (Figure 1A). On the other hand, *EMX2* expression was detected in half of ESCC and ovarian cancer cell lines but not in colon, stomach, or breast cancer cell lines, except for BT20 (Figure 1B). When the distributions of *EMX2* expression levels were visualized, they showed a uniform expression pattern in non-cancerous esophageal epithelial tissues, an all-or-none pattern in cancer cell lines, especially in ESCC cell lines, and a likely mixture of them in ESCC tissues (Figure 1C).

### 3.2. Expression of Two Isoforms, EMX2sp1 and EMX2sp2

**Figure 2:**
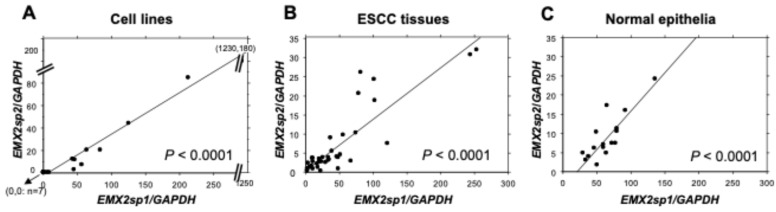
Expression of two isoforms of *EMX2*, *EMX2sp1* (splice variant 1) and *EMX2sp2* (splice variant 2). Relationship between the expression levels of *EMX2 sp1* and *sp2* evaluated by RT-qPCR in (**A**) 20 ESCC cell lines, (**B**) 37 ESCC tissue samples, and (**C**) 17 normal esophageal epithelia by relative expression levels of *EMX2* (**A**–**C**, regression analysis). (**A**): *sp2* = −2.663 + 0.37 × *sp1*, *p* < 0.0001, *R*^2^ = 0.944, when the highest was excluded as an outlier; (**B**): *sp2* = 0.135 × *sp1*, *p* < 0.0001, *R*^2^ = 0.843; (**C**): *sp2* = −4.045 + 0.199 × *sp1*, *p* < 0.0001, *R*^2^ = 0.701. Relative gene expression levels were calculated using *GAPDH* expression as the denominator for each sample (*n* = 3).

Since it is known that major splice variants of the *EMX2* gene are expressed, the relationship between them was clarified. RT-qPCR with variant-specific primer sets revealed that the levels of *EMX2* splice variant 1 (*sp1*) and variant 2 (*sp2*) were highly correlated in both groups of ESCC cell lines (Figure 2A), ESCC tissues (Figure 2B), and normal esophageal epithelial tissues (Figure 2C), and the *sp1* level was always higher than the *sp2* level (*p* < 0.0001). Spearman correlation analysis also confirmed these correlations (*p* < 0.0001—*p* = 0.0010, Figure S1).

### 3.3. Relationship Between Cell Proliferation, 5-FU Sensitivity and Colony Formation Capacity and EMX2 Expression Levels

**Figure 3:**
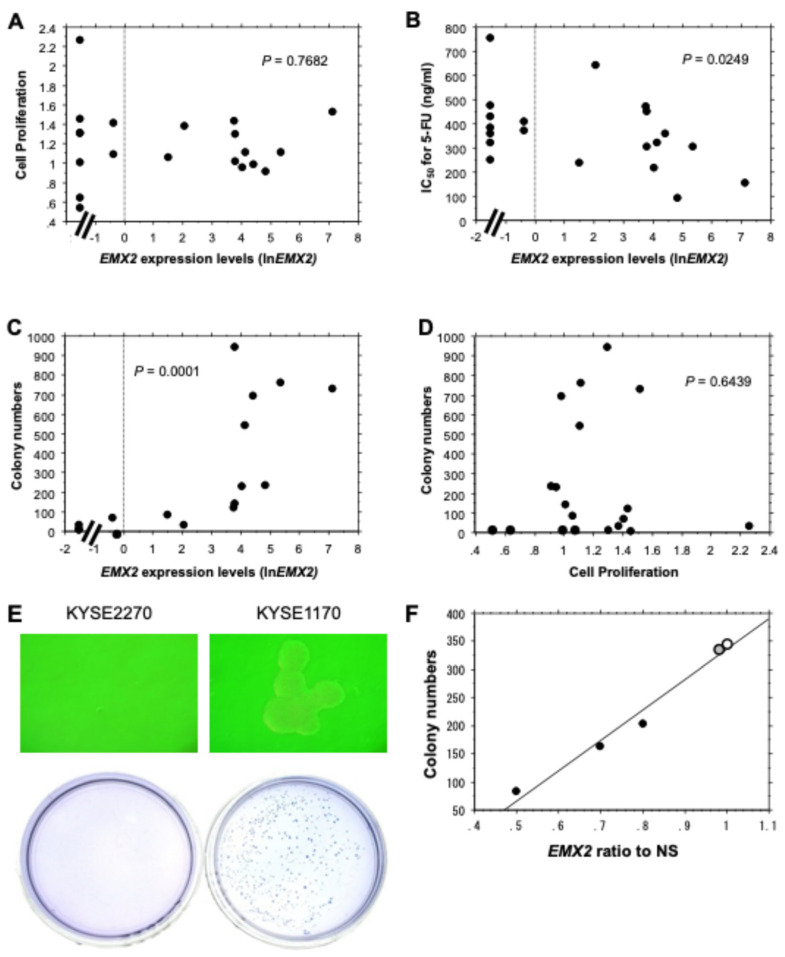
Relationship between *EMX2* expression levels in 20 ESCC cell lines and various factors. (**A**) cell proliferation capacities as assessed by the MTT assay (*n* = 3), (**B**) 5-FU sensitivity as assessed by the MTT assay (*n* = 3), and (**C**) colony formation capacities as assessed by the soft agar colony formation assay on day 14 (*n* = 3) are shown. Seven cell lines with ln*EMX2* < −1 showed no detectable *EMX2* expression. (**D**) Relationship between cell proliferation capacities and colony formation capacities are shown. (**E**) Representative example of ESCC cell line colony formation in soft agar observed by microscopic phase contrast (×100) at day 8 (top panels) and macroscopic crystal violet staining at day 14 (×10, bottom panels) are shown. KYSE1170 expressed high levels of *EMX2*, whereas KYSE2270 had no detectable *EMX2* expression. Statistical significance was calculated by Spearman’s rank correlation analysis. (**F**) Colony numbers in ESCC cell line KYSE350 transiently transfected with siRNA for *EMX2* were evaluated by the soft agar colony formation assay on day 14 (*n* = 3). Relative gene expression levels were calculated using *GAPDH* expression as the denominator for each sample (*n* = 3). The expression levels of *EMX2* in KYSE350 cells transfected with one of three types of *EMX2* siRNA (closed black circle) and the parental cells (closed gray circle) were divided by that of non-specific siRNA-transfected cells (NS, open circle).

*EMX2* expression levels evaluated by RT-qPCR in 20 ESCC cell lines showed no correlation with cell proliferation capacity (Figure 3A) but an inverse correlation with IC_50_ values to 5-FU evaluated by MTT assay (*p* = 0.0249) (Figure 3B). Soft agar colony formation assay revealed that the colony formation capacities of 20 ESCC cell lines were highly correlated with *EMX2* expression levels (*p* = 0.0001) (Figure 3C) but not with cell proliferation capacities (Figure 3D). Representative microscopic and macroscopic images showed that KYSE2270, which did not express *EMX2*, did not form any colonies, whereas KYSE1170, which expressed *EMX2*, formed many colonies (Figure 3E). Importantly, the number of colonies was decreased by siRNA-mediated knockdown of *EMX2* in a dose-dependent manner (*p* = 0.0137, *R*^2^ = 0.977) (Figure 3F), although cell proliferation capacities were not associated with *EMX2* expression levels or with the colony formation capacities described above.

### 3.4. Relationship Between EMX2 and the Expression Levels of TERT, PCNA and PROM1

**Figure 4:**
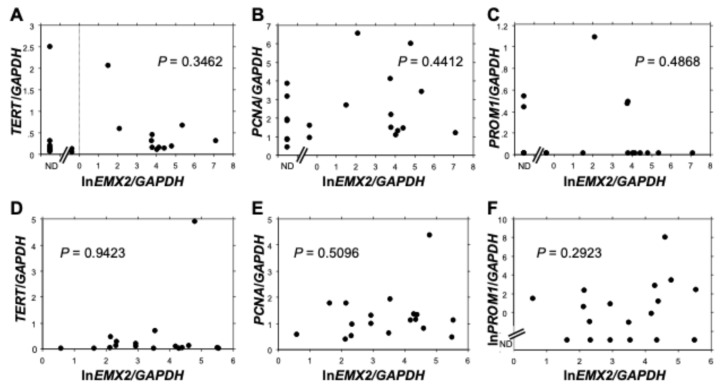
Relationship between the expression levels of *EMX2* and *TERT*, *PCNA*, and *PROM1* in (**A**–**C**) 20 ESCC cell lines and (**D**–**F**) 18 ESCC tissue samples. *p*-values were calculated by Spearman’s rank correlation analysis. ND: not detected. Relative gene expression levels were calculated using *GAPDH* expression as the denominator for each sample (*n* = 3).

Since the expression levels of *EMX2* were correlated with colony formation capacity, the expression of stemness and cell proliferation-related genes were evaluated by RT-qPCR. As a result, *EMX2* expressions did not show any correlation with those of *TERT* (encoding telomerase reverse transcriptase) (Figure 4A,D), *PCNA* (encoding proliferating cell nuclear antigen) (Figure 4B,E), or *PROM1* (encoding CD133, a well-known cancer stem cell marker) (Figure 4C,F) in either 20 ESCC cell lines or 18 ESCC tissue samples. 

### 3.5. Colony Formation Capacity of the Side Population in EMX-Expressing ESCC Cell Line

**Figure 5:**
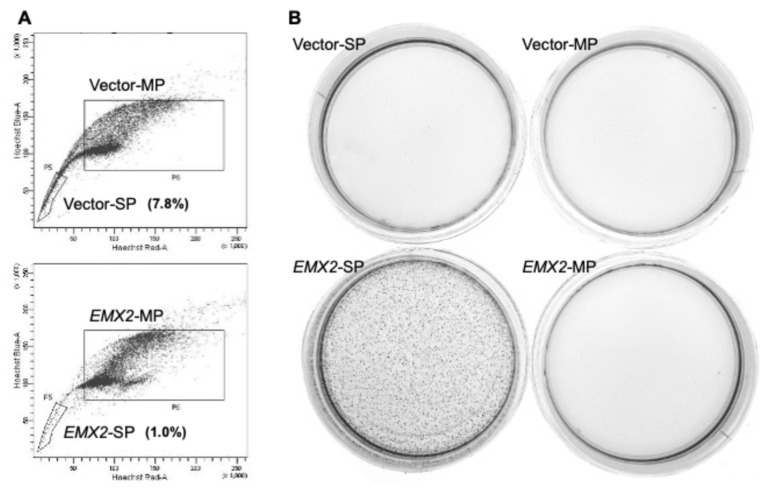
The colony formation capacity of the side population (SP) and main population (MP) fractions of KYSE510 cells with vector control (3xFLAG, top) or *EMX2* (bottom) transfection. (**A**) The SP and MP fractions were sorted and purified from each stable transfectant through Hoechst red and blue filters using FACSAria. (**B**) Each fraction was subjected to soft agar colony formation assay and macroscopically evaluated by crystal violet staining on day 14 (×10) (*n* = 3).

To clarify the role of *EMX2* expression in stemness, the ratio of side population (SP) fractions and colony formation capacity in the fraction were evaluated. KYSE510, which does not express *EMX2*, was transfected with a control vector (Vector) or EMX2 expression plasmid vector (EMX2). Cell sorting analysis by FACSAria showed that the SP fraction in EMX2-transfected KYSE510 (*EMX2*-KYSE510) (1.0%) was decreased compared to Vector-KYSE510 (7.8%) (Figure 5A). Soft agar colony formation assay revealed that only the SP fraction of *EMX2*-KYSE510 showed colony formation capacity, but the main population (MP) of *EMX2*-KYSE510, both SP and MP fractions of Vector-KYSE510 did not, suggesting a unique function of EMX2 in stemness (Figure 5B).

### 3.6. Transplantation into Non-Obese Diabetic (NOD) Scid Mice

The 10^6^ or 10^3^ *EMX2*-transfected ESCC cells were injected subcutaneously into immunodeficient mice, and tumor development was evaluated on day 32. Four independent *EMX2*-transfected ESCC clones (*EMX2*-KYSE170-1, *EMX2*/*GAPDH* = 6557; *EMX2*-KYSE170-2, *EMX2*/*GAPDH* = 9574; *EMX2*-KYSE170-3, *EMX2*/*GAPDH* = 4879; *EMX2*-KYSE510, *EMX2*/*GAPDH* = 28659) were derived from two originally *EMX2*-absent ESCC cell lines. All four *EMX2*-transfected ESCC clones of 10^6^ cells and one clone (*EMX2*-KYSE170-1) of 10^3^ cells developed tumors, while none of the two *EMX2*-absent control clones of 10^6^ cells (*EMX2*/*GAPDH* = 0), which were generated by transfection of control vector into the corresponding two ESCC cell lines, formed tumors at this time point (Figure S2). Although the EMX2-transfected KYSE510 cell-derived tumor showed a duct-like configuration, the origin of these cells was confirmed as squamous cell carcinoma by positive p63/CK14 immunostaining, except for the limited cells forming the ducts, possibly due to dysdifferentiation (Figure S3).

### 3.7. Prognosis of Patients from Whom the ESCC Cell Lines or ESCC Tissue Samples Were Derived Evaluated by Kaplan–Meier Survival Analysis

**Figure 6:**
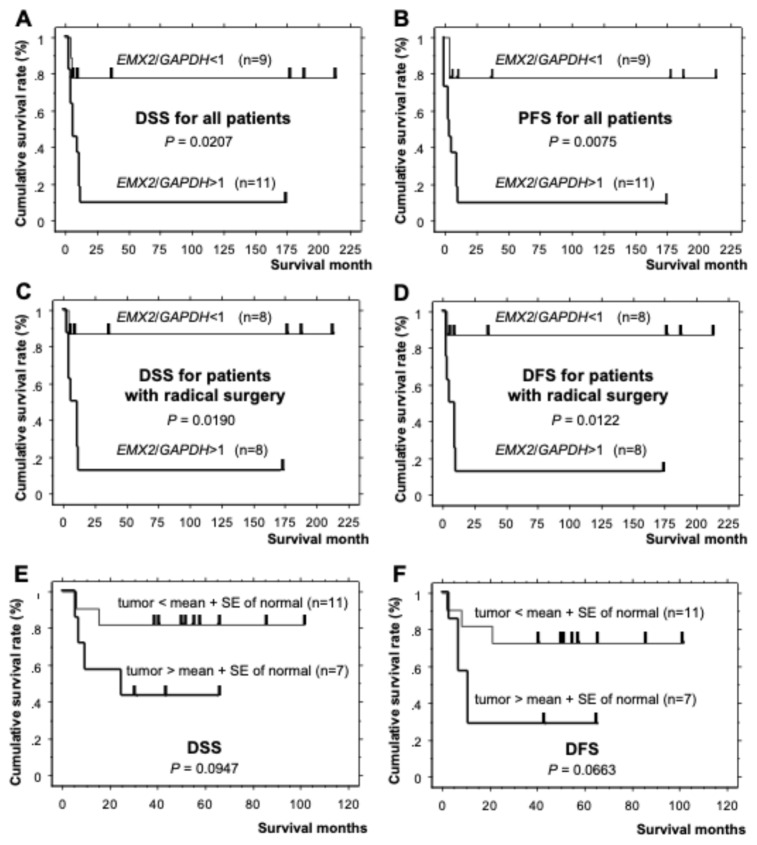
The prognosis of patients from whom the ESCC cell lines or tissue samples were derived was evaluated using a Kaplan–Meier survival analysis. The cases were divided according to *EMX2* expression levels, and (**A**,**C**) disease-specific survival (DSS), (**B**) progression-free survival (PFS), and (**D**) disease-free survival (DFS) were compared between groups. Patients were stratified by *EMX2* expression levels in their ESCC tissues. (**E**) Disease-specific survival (DSS) and (**F**) disease-free survival (DFS) after radical surgery were then compared between groups. Relative gene expression levels were calculated using *GAPDH* expression as the denominator for each sample (*n* = 3).

The prognosis of the patients from which the ESCC cell lines with high *EMX2* expression had been established was significantly worse than that of other patients from which the ESCC cell lines with nil/minimal *EMX2* expression had been established, both in terms of disease-specific survival (Figure 6A,C, DSS, *p* = 0.0207 and 0.0190 for all 20 patients and 16 patients with radical surgery, respectively), progression-free survival (Figure 6B, PFS, *p* = 0.0075 for all 20 patients), and disease-free survival (Figure 6D, DFS, *p* = 0.0122 for 16 patients with radical surgery). This trend was also observed in ESCC tissue samples (Figure 6E,F, *p* = 0.0947 and 0.0663 for DSS and DFS, respectively, in 18 patients).

## 4. Discussion

Our study highlights the critical role of Empty Spiracles Homeobox 2 (EMX2) in the progression and prognosis of esophageal squamous cell carcinoma (ESCC). We demonstrated that *EMX2* expression is significantly reduced in ESCC tissues compared to adjacent normal esophageal epithelium. In addition, our findings suggest that EMX2 functions as a key regulator of tumor stemness in a specific part and chemotherapeutic sensitivity, with high *EMX2* expression correlating with increased colony formation capacity in vitro, tumor formation capacity in vivo, and reduced sensitivity to 5-FU treatment.

The observed downregulation of *EMX2* in ESCC tissues is consistent with reports in other malignancies where *EMX2* functions as a tumor suppressor gene. For example, *EMX2* downregulation has been associated with poor prognosis in glioblastoma [12], ovarian cancer [19], and lung cancer [20]. *EMX2* is a transcription factor that has been studied in the development of the central nervous system [9,10,21,22]. In addition, when it was first identified as a homeodomain gene, a homolog of Drosophila melanogaster empty spiracles, in the deletion interval of endometrial cancer at 10q25.3-26.1, it was suggested to be a candidate tumor suppressor gene, inversely correlated with endometrial proliferation [23]. Taken together, EMX2 may have a unique function in cancer cells other than that of a tumor suppressor or oncogene. Interestingly, our results indicate that EMX2 decreases the proportion of side population (SP) of ESCC cells but may increase cancer stemness within this fraction, as evidenced by the increased colony formation capacity in EMX2-expressing cells. These findings are consistent with previous studies suggesting that homeobox genes regulate cellular differentiation and tumor plasticity [11]. Additionally, it has been reported that the stem cell/progenitor gene program and the Paneth cell maturation program, both of which are driven by Wnt signaling in the crypt, are activated in intestinal cancer [24]. As EMX2 has been suggested to regulate the Wnt signaling pathway [22,25,26], further elucidating the molecular mechanisms of EMX2 function is desirable.

The relationship between *EMX2* expression and 5-FU resistance is particularly interesting. Our results show a correlation between *EMX2* levels and 5-FU sensitivity (inversely correlated with IC_50_ values to 5-FU), suggesting that decreased expression of EMX2 may contribute to chemoresistance in ESCC. This finding parallels report in other cancer where homeobox genes influence drug sensitivity by modulating apoptotic pathways and drug efflux mechanisms [20]. Further studies are warranted to elucidate the molecular mechanisms underlying EMX2-mediated chemosensitization.

Since two isoforms of *EMX2* mRNA, *sp1* consisting of three exons and *sp2* lacking exon 2, have been registered at EMBL-EBI (ENSG00000170370), each form-specific RT-qPCR was designed in this study. The amounts of *sp1* mRNAs were always highly correlated with those of *sp2* and higher than the latter, so that *sp1* expression levels were considered to be recognized as *EMX2* expression levels. Since the functional differences between *sp1* and *sp2* isoforms are still unknown, they may be crucial for a unique function of EMX2 in cancer cells. A uniform expression pattern was also observed in non-cancerous esophageal epithelial tissues, an all-or-none pattern in ESCC cell lines, and probably a mixture of these in ESCC tissues. The reason for the complex expression pattern in ESCC tissues is still unclear, but it may be due to contamination from non-cancerous tissues or a silencing mechanism during immortalization (to become a cell line). Furthermore, the prognostic impact of *EMX2* expression was importantly observed in both ESCC cell lines and ESCC tissues, suggesting that the *EMX2* expression status was not altered during cell line establishment, representing a higher malignant potential of ESCC.

Although preliminary, our in vivo transplantation experiments in NOD-*scid* mice further validate the oncogenic potential of EMX2 in ESCC, although it is still preliminary. *EMX2*-transfected ESCC cells consistently developed tumors, whereas control vector-transfected ESCC cells failed to form tumors under similar conditions. These results strongly suggest that *EMX2* expression enhances tumorigenicity, possibly by promoting stem-like characteristics and self-renewal capacity. FACS analysis showed that *EMX2* expression reduced the proportion of side population (SP) cells while increasing the clonogenic potential within this fraction. This suggests that while EMX2 may not directly regulate the size of the cancer stem-like population, it enhances the functional properties required for tumor initiation and progression.

The prognostic significance of *EMX2* in ESCC is underscored by our Kaplan–Meier survival analyses, which showed that patients with high *EMX2* expression have significantly worse disease-specific survival (DSS), progression-free survival (PFS), and disease-free survival (DFS). To compensate for the limited power of our small-scale analysis, we performed additional analyses using the public database Kaplan–Meier Plotter. The results confirmed our findings, demonstrating a significant association between higher *EMX2* expression and relapse-free survival (RFS) in esophageal squamous cell carcinoma (ESCC) (*n* = 81, HR = 2.71, log-rank *p* = 0.042, Figure S4). Furthermore, similar results were obtained in patients with head and neck squamous cell carcinoma (HR = 4.44, log-rank *p* = 0.0026, Figure S4) and cervical squamous cell carcinoma (HR = 2.37, log-rank *p* = 0.025, Figure S4), suggesting that this phenomenon may be more widespread in SCC. These findings support the notion that *EMX2* expression serves as a potential biomarker for poor prognosis in ESCC. While the precise regulatory mechanisms governing *EMX2* expression in ESCC remain unclear, epigenetic modifications such as promoter methylation and histone modifications may play a role, as suggested by studies in esophageal, glioblastoma, and ovarian cancers [12,21,27]. The functional significance of EMX2 in poor prognosis in ESCC also remains unclear. *EMX2* expression was correlated with colony formation capacity in soft agar but not with cell proliferation capacity and expressions of genes related to cell proliferation, immortalization, and cancer stem cells, suggesting that the poor prognosis of ESCC patients with high *EMX2* expression cannot be explained by rapid growth, immortalization, or existence of cancer stem cells, but something in cancer stemness phenotype. 

In conclusion, our study provides compelling evidence that EMX2 plays a pivotal role in ESCC progression by enhancing cancer stemness and correlating with poor patient prognosis. These findings highlight the potential of EMX2 as a novel prognostic biomarker and therapeutic target for ESCC. Future studies should focus on elucidating the upstream regulators of EMX2 and exploring targeted strategies to modulate its expression for therapeutic benefit.

## Data Availability

The original contributions presented in the study are available in the article/Supplementary Material; for further information, please contact the corresponding author.

## Supplementary Materials

The following supporting information can be downloaded at https://www.mdpi.com/article/10.3390/biomedicines13061373/s1: Table S1: Primer and probe set for RT-qPCR; Table S2: siRNA for knockdown experiment; Table S3: Primer set for PCR; Figure S1: Expression of two isoforms of *EMX2*, *EMX2sp1* (splice variant 1) and *EMX2sp2* (splice variant 2); Figure S2: Tumor formation in immunodeficient mice by *EMX2-*transfected ESCC cells and EMX2 expression in these cells; Figure S3: Histopathology of the tumors developed in NOD-*scid* mice; Figure S4: Prognostic significance of *EMX2* expression in patients with squamous cell carcinomas.
