# Supplementary material for: Unique Function in Cancer Stemness and Prognostic Significance of EMX2 in Esophageal Squamous Cell Carcinoma
Source: Biomedicines. 2025 Jun 4;13(6):1373. doi: 10.3390/biomedicines13061373 (PMC12190849; doi:10.3390/biomedicines13061373)
Supplement: Supplementary file 1 [file biomedicines-13-01373-s001.zip › biomedicines-3532530-supplementary.pdf]

biomedicines

MDPI

Supplementary Table S1

Primer and probe set for RT-qPCR

EMX2sp1	F: AGCCTCACGGAAACTCAGG
	R: TTGCGAATCTGAGCCTTCTT
	Probe: UPL probe: #29
EMX2sp2	F: ATCGCTTCCAAGGTAAAAGT
	R: TTGCGAATCTGAGCCTTCTT
	Probe: UPL probe: #29

Supplementary Table S2

siRNA for knockdown experiment

EMX2-specific siRNA:

ID# 107312: sense: GGGACGCACCAUAUUAACCtt,

antisense: GGUUAAUAUGGUGCGUCCCtt,

ID# 107313: sense: GGUAUUCCGUUUAUCACAGtt,

antisense: CUGUGAUAAACGGAAUACCtg,

ID# 107314: sense: GGCUUAAAACCCUGAUGCAtt,

antisense: UGCAUCAGGGUUUUAAGCCtc)

Negative control siRNA:

Silencer® Negative Control siRNA

Supplementary Table S3

Primer set for PCR

EMX2sp1	F: GGAATTCCATGTTCCAGCCGGCGCCCAA
	R: GAAGATCTCCATGTTGTCCGTTTCTGTGGG

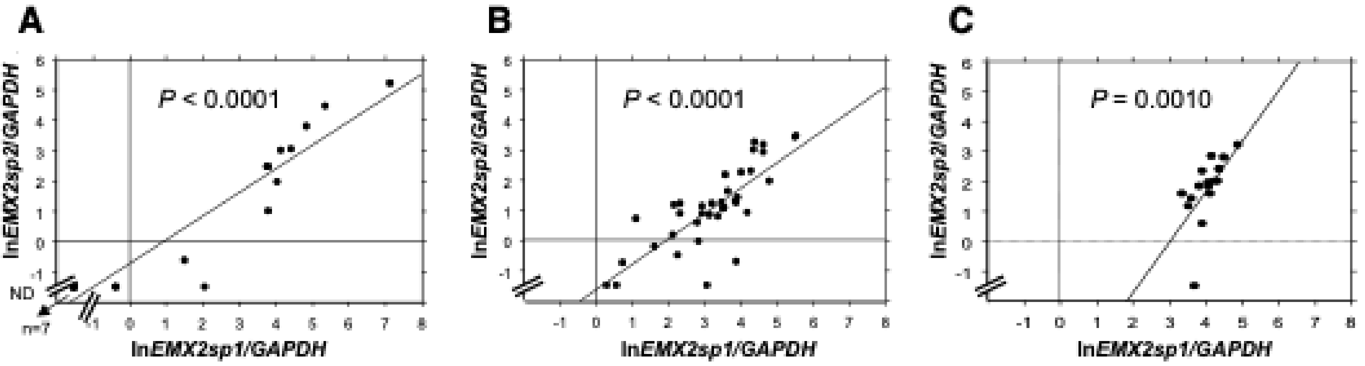

FigureS1. Expression of two isoforms of *EMX2*, *EMX2sp1* (splice variant 1) and *EMX2sp2* (splice variant 2). Relationship between the expression levels of *EMX2 sp1* and *sp2* evaluated by RT-qPCR in (A) 20 ESCC cell lines, (B) 37 ESCC tissue samples, and (C) 17 normal esophageal epithelia by relative expression levels of ln*EMX2* (Spearman rank correlation analysis). ND: not detected. Relative gene expression levels were calculated using *GAPDH* expression as the denominator for each sample (n = 3).

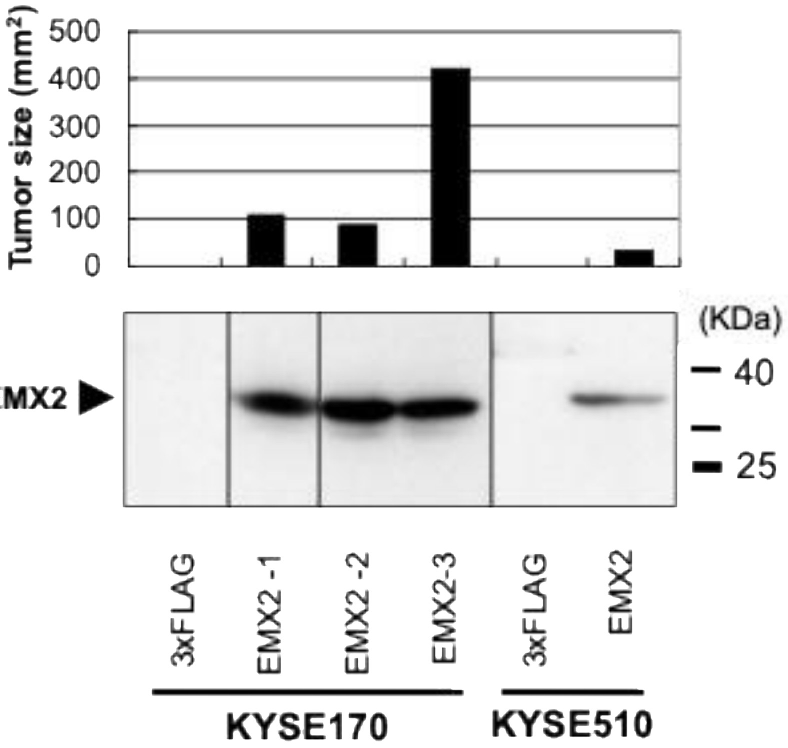

Figure S2. Tumor formation in immunodeficient mice by *EMX2*-transfected ESCC cells (top) and EMX2 expression in these cells (bottom). A total of 1 million and 10^3 cells of *EMX2*-transfected (n = 4) and control vector-transfected (n = 2) ESCC cell clones derived from two originally *EMX2*-absent ESCC cell lines, KYSE170 and KYSE510, were injected s.c. at a total of 18 sites in the shoulder and flank of five mice. All clones with *EMX2*-transfection in 2 types of ESCC cell lines developed tumors on day 32 after subcutaneous injection of 10^6 cells (EMX2-1 - 3, EMX2), whereas no tumor was found from the KYSE170 and KYSE510 cells transfected with control vector (3xFLAG). Tumor size is represented by the long diameter x short diameter (mm): 12 x 9, 10 x 9, 28 x 15, and 11 x 3, respectively. Western blotting conditions: 1st antibody: anti-FLAG M2 1:2000, 2nd antibody: mouse Ig HRP 1:5000, Whole-cell extract: 25μg/each sample.

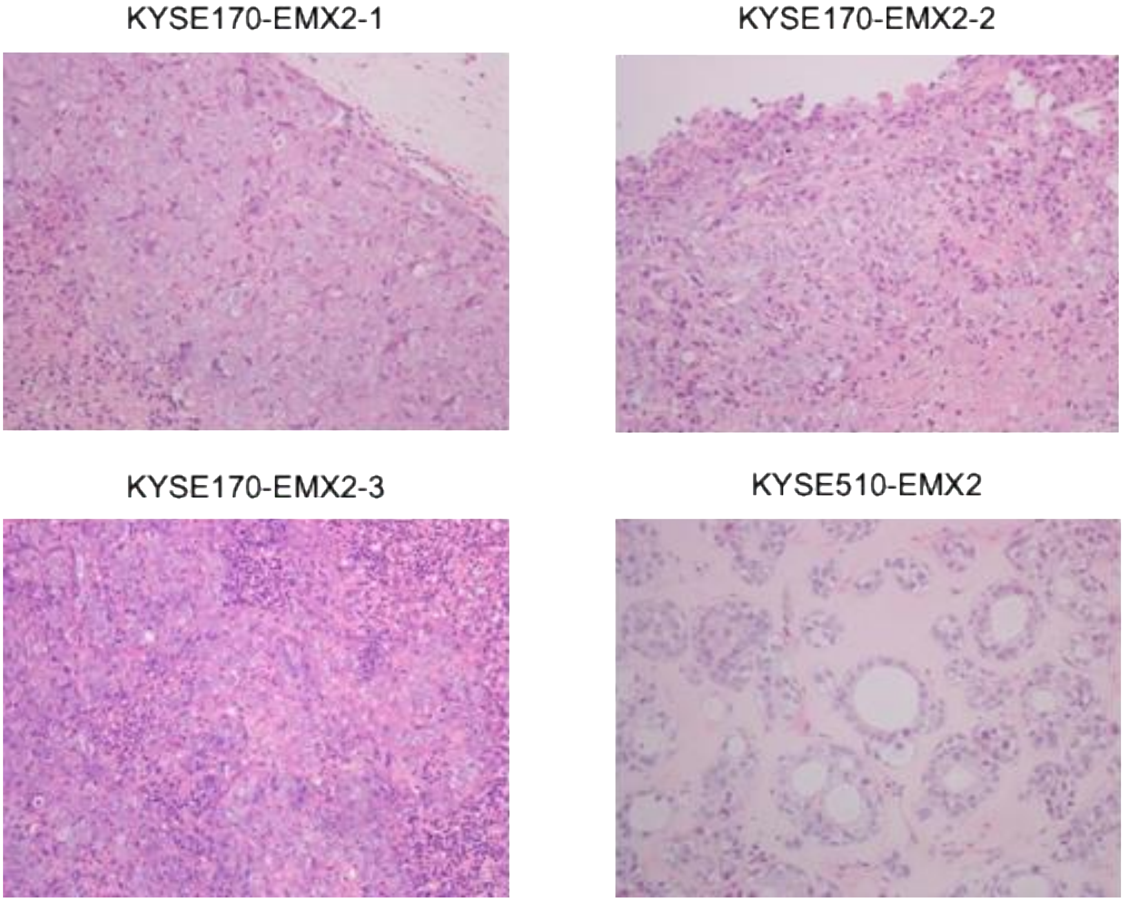

Figure S3. Histopathology of the tumors developed in NOD-*scid* mice. All 4 independent *EMX2*-transfected ESCC clones derived from non-*EMX2* expressing ESCC cell lines KYSE170 and KYSE510 formed tumors on day 32 after subcutaneous injection of 10^6 cells (EMX2-1 - 3, EMX2), whereas both vector control transfected clones did not form tumors at this time point. Although the tumor derived from EMX2-transfected KYSE510 cells (bottom right) showed a duct-like configuration, the origin of these cells was confirmed as squamous cell carcinoma by positive p63/CK14 immunostaining, except for the limited cells forming the ducts, possibly due to dysdifferentiation. Microscopic images were taken at x200 magnification.

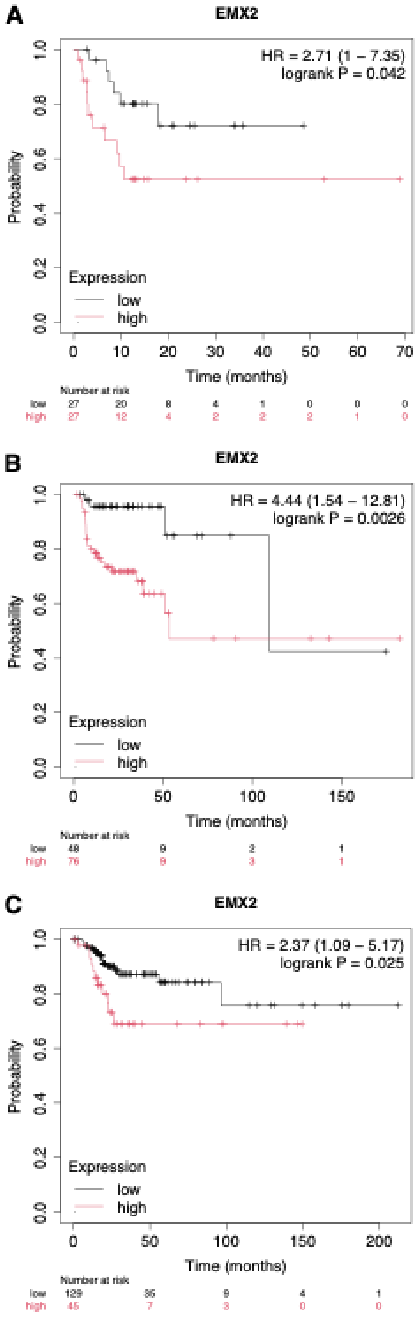

Figure S4. Prognostic significance of *EMX2* expression in patients with squamous cell carcinomas. A meta-analysis-based validation was performed using the Kaplan–Meier Plotter (http://kmplot.com/analysis/). Progression-free survival (PFS) was compared between patients with (A) esophageal, (B) head and neck, and (C) cervical squamous cell carcinomas who expressed high or low levels of *EMX2*. The hazard ratio (HR) and log-rank *P*-value are indicated in each panel.
